# Buffalo Physicians in the Government Service

**Published:** 1862-06

**Authors:** 


### BUFFALO PHYSICIANS IN THE GOVERNMENT SERVICE.

We renew the list published some time since of Buffalonians and graduates from the College here, who are in the government service, as there have been additions and changes since that time.

U. S. ARMY.

Dr. Chas. K. Winne, - - - Ass't Surg., Clarksburgh. Va.

VOLUNTEERS.

Dr. Chas. H. Wilcox, - - Brigade Surg., Genl. Patrick's Brigade.
(Resigned, and is again Surgeon 21st Reg't N. Y, V.)
" Jos. A. Peters, - - Ass't Surg., 21st N. Y. V
" Lucien Damainville, - Surgeon 31st N. Y. V.
" E. P. Gray, - - - Surgeon 100th Regt,, N. Y. V.
" E. L. Bissell, - - Ass't Surgeon 44th N. Y. V.
" J. W. Casey, - - - " " 105th N. Y. V.
" Wm. H. Butler, (Sick leave.) " " Mich. Volunteers.

| | | |
|---|---|---|
| Dr. F. J. Bancroft, - - | Ass't Surgeon Penn, Volunteers. | |
| " Sylvester Rankin, - - | " " | New Mexico Volunteers. |
| " S. B. Hunt, - - - - | Volunteer Surgeon, Yorktown. | |
| " Charles Winne, - - | " " | |

U. S. NAVY.

| | | |
|---|---|---|
| Dr. Newton N. Bates, - - | Ass't Surgeon, | Gunboat "Seneca." |
| " Wm. Howell, - - - | " " | Sick leave |
| " S. D. Flagg, Jr. - - | " " | Gunboat "Connecticut." |
| " Wm. B. Mann, - - - | " " | Gunboat "Miami." |
| " Geo. D. Slocum. - - | " " | S. Sloop "San Jacinto." |
| " H. P. Babcock, - - - | " " | Waiting Orders. |

U. S. VOLUNTEER NAVY.

| | | |
|---|---|---|
| Dr. Ira C. Whitehead. - - | Ass't Surgeon, | Key West. |
| " Geo. L. Sweet, - - | " " | Gunboat "Isaac Smith." |

WITH THE SANITARY COMMISSION.

| | |
|---|---|
| Dr. Horace Tupper, - - - - - | Pittsburgh Landing. |
| " C. B. Hutchins, - - - - - - - | " " |

